# Quantification of Lung Fibrosis in IPF-Like Mouse Model and Pharmacological Response to Treatment by Micro-Computed Tomography

**DOI:** 10.3389/fphar.2020.01117

**Published:** 2020-07-21

**Authors:** Francesca Ruscitti, Francesca Ravanetti, Valeria Bertani, Luisa Ragionieri, Laura Mecozzi, Nicola Sverzellati, Mario Silva, Livia Ruffini, Valentina Menozzi, Maurizio Civelli, Gino Villetti, Franco Fabio Stellari

**Affiliations:** ^1^ Corporate Pre-Clinical R&D, Chiesi Farmaceutici S.p.A., Parma, Italy; ^2^ Department of Veterinary Science, University of Parma, Parma, Italy; ^3^ Department of Medicine and Surgery, University of Parma, Parma, Italy; ^4^ Department Nuclear Medicine, Academic Hospital of Parma, Parma, Italy

**Keywords:** lung fibrosis, animal model, mice, bleomycin, micro-CT, Nintedanib, idiopathic pulmonary fibrosis, drug discovery

## Abstract

Idiopathic pulmonary fibrosis (IPF) is a chronic progressive degenerative lung disease leading to respiratory failure and death. Although anti-fibrotic drugs are now available for treating IPF, their clinical efficacy is limited and lung transplantation remains the only modality to prolong survival of IPF patients. Despite its limitations, the bleomycin (BLM) animal model remains the best characterized experimental tool for studying disease pathogenesis and assessing efficacy of novel potential drugs. In the present study, the effects of oropharyngeal (OA) and intratracheal (IT) administration of BLM were compared in C57BL/6 mice. The development of lung fibrosis was followed *in vivo* for 28 days after BLM administration by micro-computed tomography and *ex vivo* by histological analyses (bronchoalveolar lavage, histology in the left lung to stage fibrosis severity and hydroxyproline determination in the right lung). In a separate study, the antifibrotic effect of Nintedanib was investigated after oral administration (60 mg/kg for two weeks) in the OA BLM model. Lung fibrosis severity and duration after BLM OA and IT administration was comparable. However, a more homogeneous distribution of fibrotic lesions among lung lobes was apparent after OA administration. Quantification of fibrosis by micro-CT based on % of poorly aerated tissue revealed that this readout correlated significantly with the standard histological methods in the OA model. These findings were further confirmed in a second study in the OA model, evaluating Nintedanib anti-fibrotic effects. Indeed, compared to the BLM group, Nintedanib inhibited significantly the increase in % of poorly aerated areas (26%) and reduced *ex vivo* histological lesions and hydroxyproline levels by 49 and 41%, respectively. This study indicated that micro-computed tomography is a valuable *in vivo* technology for lung fibrosis quantification, which will be very helpful in the future to better evaluate new anti-fibrotic drug candidates.

## Introduction

Idiopathic pulmonary fibrosis (IPF) is a multifactorial disease that is characterized by progressive worsening in lung function, an unfavorable prognosis and subsequent death for respiratory failure (Martinez et al., 2017). Continued research efforts are needed to identify better and innovative pharmacological treatments, since there is little evidence from randomized clinical trials of disease progression retardation with current medications and only lung transplantation prolongs the survival (Varone et al., 2017; Kumar et al., 2018).

Many research groups are committed to develop new drugs for IPF patients and to improve the predictivity of preclinical animal models, but translation into the clinic is strongly linked to the capability of these animal models to reflect human physiology, pathobiology of the disease and response to treatment. Different mouse models have become available over the years, but no one seems to be able to fully reproduce the features of IPF. This, however, should not underestimate the fact that animal models are essential prerequisites for revealing molecular mechanisms involved in the pathogenesis of lung fibrotic processes and the subsequent development and validation of prognostic tests and therapeutic interventions.

The bleomycin (BLM)-induced pulmonary fibrosis mouse model is the most used and internationally recognized animal model (Jenkins et al., 2017). BLM has an important role in chemotherapy for the treatment of some forms of cancers, such as germinative tumors and Hodgkin’s lymphoma. However, its use can be life-threatening in up to 10% of the patients receiving the drug, since the treatment is associated with the occurrence of interstitial pulmonary fibrosis (also called fibrosing alveolitis) (Balikian et al., 1982; Calaway et al., 2018). This evidence led to BLM use in experimental models and has allowed the replication of some key hallmarks of IPF, such as damage to alveolar epithelial cells, fibroblast and myofibroblast activation and collagen deposition (Scotton and Chambers, 2010; Liu et al., 2017; Carrington et al., 2018).

Current outcome measurements in the BLM and other experimental lung fibrosis models involve labor-intensive biochemical analysis and/or histological scoring. Moreover, these invasive read-outs, such as hydroxyproline lung tissue content, histo-morphometric quantification of collagen deposition and fibrotic masses accumulation in the parenchyma surrounding the airways, require the animal sacrifice at designed time points, precluding longitudinal studies. In humans, longitudinal characterization of lung fibrotic diseases extent and progression by noninvasive imaging techniques, such as high-resolution computed tomography (HRCT) (Lynch et al., 2018; Raghu et al., 2018; Sverzellati et al., 2018; Wu et al., 2018), magnetic resonance imaging (MRI) (Weatherley et al., 2018; Wild, 2018), and positron emission tomography (PET) (Justet et al., 2017; Win et al., 2018), is now well established. Recently, these techniques with the prefix of “Micro” (CT, PET and MRI) have been optimized and validated to assess lung fibrosis at different time points in living animals and so that each subject can act as their own control (Egger et al., 2013; Egger et al., 2014; Withana et al., 2016; Désogère et al., 2017; Ruscitti et al., 2017; Ruscitti et al., 2018).

In our laboratory a mouse model of lung fibrosis induced by double BLM intratracheal instillation was recently used for primary screening showing some limitations such as a patchy fibrosis distribution through the lobes (Ruscitti et al., 2017; Stellari et al., 2017).

In 2017 the American Thoracic Society workshop report confirmed a consensus view on the murine intratracheal (IT) BLM model for the preclinical testing of antifibrotic compounds, but the panel of experts suggested space for model refinement and proposed the oropharyngeal aspiration (OA) as a valuable alternative to IT BLM instillation.

In line with this suggestion, the first objective of the present work was to compare IT and OA double BLM administration. The OA model was then explored in the second part of the work. After the set-up phase, the pharmacological validation was performed using Nintedanib, an FDA-approved drug for IPF treatment, and micro-CT imaging was proposed to longitudinally assess and quantify fibrosis progression and response to treatment. *Ex vivo* measurements such as hydroxyproline content as marker of collagen deposition extracted from the right lobe and histo-morphometric measurements derived from the left lobe were finally compared to micro-CT parameters.

The overall goal was to establish a robust, reproducible, less variable mouse model, with a uniform lung fibrosis distribution, in order to resize the number of mice used for drug discovery experiments.

## Material and Methods

### Experimental Animals

Female inbred C57Bl/6 (7- to 8-week old) mice were purchased from Envigo, Italy (San Pietro al Natisone, Udine, Italy). American Thoracic Society suggests the use of male mice, since they are more susceptible to BLM treatments and should require lower BLM concentrations compared with females. However, the National Institutes of Health recommends use of both male and female animals in all research studies. We used female mice since they are much less aggressive than males and do not require frequent interventions to separate the dominant.

Prior to use, animals were acclimatized for at least 5 days to the local vivarium conditions (room temperature: 20–24°C; relative humidity: 40–70%; 12-h light–dark cycle), having free access to standard rodent chow and softened tap water. From day 0, mice were given daily high calories dietary supplement (Recovery gel from Dietgel) and sterile sunflower seeds in addition to the standard rodent chow, since in our experience this food supplementation helps in reducing body weight loss and percentage of mortality in BLM models.

All animal experiments described herein were approved by the intramural animal-welfare committee for animal experimentation of Chiesi Farmaceutici under protocol number: 449/2016-PR and comply with the European Directive 2010/63 UE, Italian D.Lgs 26/2014 and the revised “Guide for the Care and Use of Laboratory Animals” (Committee for the Update of the Guide for the Care and Use of Laboratory Animals and National Research Council, 2010).

### Oropharyngeal Administration

Animals were lightly anesthetized with 2.5% isoflurane delivered in a box and bleomycin hydrochloride [BAXTER (1 mg/kg) in 50 µl of saline (0.9%) or vehicle (50 µl of saline (0.9%)] was administered *via* oropharyngeal (OA) using a micropipette (De Vooght et al., 2009).

Mice were positioned on the intubation platform, hanging them by their incisors placed on the wire, the tongue was pulled out with forceps, using a small laryngoscope and with a micropipette the liquid was placed onto the distal part of the oropharynx while the nose was gently closed. This procedure was performed at days 0 and 4.

### Intratracheal Administration

Animals were lightly anesthetized with 2.5% isoflurane delivered in a box and bleomycin hydrochloride BAXTER (1 mg/kg) in 50 µl of saline (0.9%) or vehicle [50 µl of saline (0.9%)] was instilled directly through a tracheal cannula, using a small laryngoscope (Penn-Century Inc., Philadelphia, PA, USA) to visualize the trachea (Ruscitti et al., 2018).

As for the OA protocol, the procedure was performed at days 0 and 4.

### Nintedanib

Nintedanib, an FDA-approved drug, was dissolved in 1% of tween 80 in milliQ water at 6 mg/ml and administered at 60 mg/kg by oral gavage.

### Bronchoalveolar Lavage and Cells Count

At days 7, 14, 21 and 28 after the first bleomycin challenge, animals were anesthetized with isoflurane and sacrificed by bleeding from the abdominal aorta. Bronchoalveolar lavage fluid (BALF) was collected by gently washing the lungs with 0.6 ml sterile solution [Hank’s balanced salt solution × 10; ethylenediaminetetraacetic acid 100 mM; 4-(2-hydroxy-ethyl)-1-piperazineethansulphonic acid 1 mM; and distilled water] for three times in the bronchial tree and preserved for subsequent analysis (Stellari et al., 2015b). The cell pellet was resuspended in 0.2 ml of PBS. Cell number was counted with an automated cell counter (Dasit XT 1800J, Sysmex).

### Computed Tomography

After mice were anesthetized with isoflurane at 2%, lung imaging was performed at 7, 14, 21 and 28 days with Quantum GX Micro-CT (PerkinElmer, Inc. Waltham, MA). Images were acquired with a respiratory gated technique with the following parameters: 90 KV, 88 µA over a total angle of 360° for a total scan time of 4 min. The entire set of projection radiographs was reconstructed using a filtered back-projection algorithm with a Ram-Lak filter. The acquisition protocol in ‘high speed’ mode, resulted in two 3D datasets with 50 μm isotropic reconstructed voxel size, corresponding to two different phases of the breathing cycle. Data reported here are referred to the end of expiration phase. For each examination, a stack of 512 cross-sectional images stored in unsigned 16-bit file format was produced. The reconstructed datasets were analyzed using Perkin Elmer Analyze software (Analyze 12.0; Copyright 1986-2017, Biomedical Imaging Resource, Mayo Clinic, Rochester, MN). A semi-automatic segmentation was used to define airways and total lung volumes. For quantitative assessment of the lung parenchyma, Hounsfield Unit (HU) clinical ranges (Gattinoni et al., 2001) were applied on rescaled HU images. Airways (AW) and blood vessels (BV) were segmented and used as reference points for the alignment with 2D histological sections, and lung tissue divided in poorly- ([−500, −100] HU) and normo-aerated ([−900, −500] HU).

### Histology

Lungs were removed, inflated with a cannula through the trachea by gentle infusion with 0.6 ml of 10% neutral-buffered formalin and fixed for 24 h. For each animal, lungs were divided in five lobes (right: cranial, middle, caudal, accessory lobe and left lobe); these have been dehydrated in graded ethanol series, clarified in xylene and paraffin embedded. Three serial sections 5 μm thick were obtained at 200 μm intervals, using a rotary microtome (Slee Cut 6062, Slee Medical, Mainz, Germany), following the dorsal plane. Sections were stained with Masson’s trichrome standard protocol (Histo-Line Laboratories). For analysis, whole-slide images were acquired by NanoZoomer S-60 Digital slide scanner (NanoZoomer S60, Hamamatsu, Japan). Fibrotic lung injury was assessed histologically through Ashcroft scoring system (Hübner et al., 2008) by two independent researchers blinded to the experimental design. For Ashcroft score analysis the whole lung parenchyma was considered for the analysis at the magnification of 20× after slide digitalization. To improve the Ashcroft score informativeness, the point score was subdivided into three major categories: mild (0–3), moderate (4) and severe (5–8); their frequency distribution was then analyzed (Stellari et al., 2017). In the pharmacological experiment with Nintedanib, the left lobe was fixed and used for histological analysis and right lobes for the hydroxyproline quantification.

### Biochemical Quantification of Hydroxyproline

The content of hydroxyproline in right lung was determined using a commercial kit from Sigma-Aldrich according to the manufacturer’s protocol. Briefly the right lobes were homogenized in PBS and an aliquot was hydrolyzed in 6 N HCl for 24 h at 100°C. Then, hydroxyproline concentration was determined by the reaction of oxidized hydroxyproline with 4-(Dimethylamino) benzaldehyde (DMAB), which resulted in a colorimetric (560 nm) product, proportional to the hydroxyproline present. The total amount of hydroxyproline was calculated on the right lobes weight.

### Statistics

All data are presented as mean ± s.e.m. or SD. Statistical analysis was performed on raw data using one-way analysis of variance (ANOVA), followed by Dunnett’s t post-hoc test or Tukey’s test. If data were not normally distributed, Kruskall–Wallis test was performed followed by Dunn’s multiple comparison test. Statistical analysis was performed using GraphPad software, version 7.0. P <0.05 was considered a level of statistical significance.

## Results

The direct comparison between IT and OA double BLM administration was the first part of this study.

Eighty-four mice in total were either IT or OA treated at day 0 and at day 4, with BLM or saline as control group. All the mice were visually monitored daily and weighed over the course of the study. BLM administration caused body weight loss of about 10% with a maximal reduction from days 9 to 14, nevertheless no mortality has been observed for both protocols; no changes in body weight were noticed in the control group (**Supplementary Figure 1A**).

A significant increased number of white blood cells (WBC) in BALF has been found at 7 and 14 days in both IT and OA models (**Supplementary Figure 1B**).

Differential cell analysis of BALF revealed that macrophages and lymphocytes are the two prevalent populations both after IT and OA BLM administration. Specifically, the time course of macrophages infiltration followed a similar trend after both IT and OA administration, peaking at days 7 and 14 and then declining or returning to baseline as monitored to day 28 (**Supplementary Figure 1C**). A different kinetic of recruitment has been observed for lymphocytes, which increased later at day 14 and were still significantly elevated at day 21 for the IT protocol only (**Supplementary Figure 1D**). The peak in neutrophils numbers occurred at days 7 and 14, after IT BLM, coinciding with the acute inflammatory reaction induced by BLM: the increase was short-lasting in the BLM OA group, being significant only at day 7 (**Supplementary Figure 1E**). In summary, no relevant differences can be highlighted comparing the protocols.

**Figure 1:**
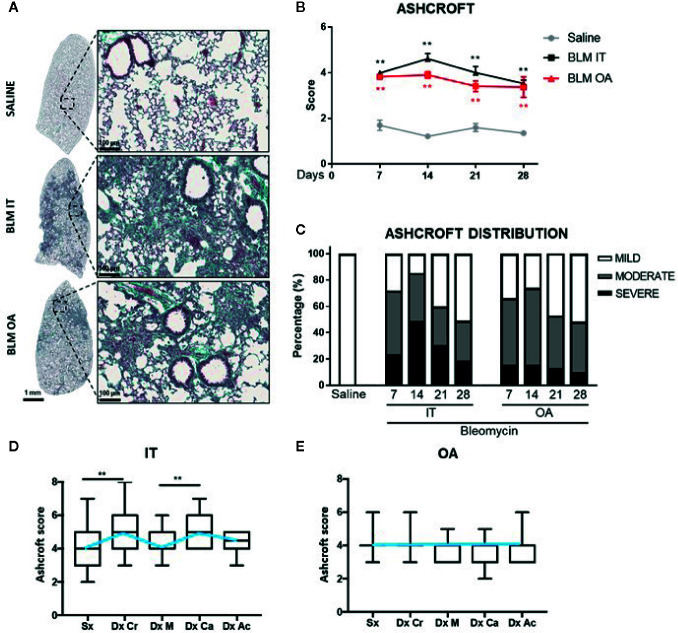
Representative whole slide images of Masson’s trichrome stained section of the left lung with enlarged details (scale bar 100 μm) at 14 days after BLM treatment with IT or OA protocols and saline respectively **(A)**. The total fibrosis quantification based on Ashcroft **(B)**. The data represent the mean ± s.e.m. of seven animals for each time point per group. Changes in BLM groups were compared to the saline group using two-way ANOVA followed by Dunnett’s test. *p <0.05; **p <0.01. Time course of frequency distribution of Ashcroft scores divided in three classes as (1–3) mild, 4 moderate, ≥5 severe fibrosis after BLM treatment with IT or OA protocols and saline respectively **(C)**. Ashcroft score among the five lobes at 14 days for IT **(D)** and OA BLM administration **(E)**. Left lobe (Sx); right: cranial (Dx Cr), middle (Dx M), caudal (Dx Ca), accessory lobe (Dx Ac). Data distribution of seven animals for each time point per group are graphed as box plot and the median values are linked through a light blue line. Kruskall–Wallis test was performed followed by Dunn’s multiple comparison test to compare Ashcroft score among the lung lobes. *p <0.05; **p <0.01.

Representative histological left lobes sections of saline and IT and OA BLM-treated mice at day 14 are shown in **Figure 1A** as whole slide images and enlarged details. The histological analysis of the lung parenchyma revealed a marked heterogeneity in the fibrosis distribution among various lobes for all time points of observation in BLM IT compared to OA treated mice (**Figure 1D** and **Supplementary Figure 2**). The lung parenchyma modifications ranged from isolated gentle fibrotic changes up to contiguous fibrotic walls, more evident in IT compared to OA BLM group. In the IT protocol, alveolar space alterations varied from partly enlarged air spaces to partial and complete obliteration due to fibrotic proliferation (**Figure 1A** and **Supplementary Figure 3**), while the fibrotic lesions in OA-treated mice occurred as diffuse single fibroproliferative loci. At day 28, an initial tendency towards resolution appeared with both protocols (**Supplementary Figure 3**). Fibrosis in OA treated mice appeared more homogeneous, less variable among animals and uniformly distributed throughout the lobes at 14 days (**Figure 1E**), as well as at other time points of observation (**Supplementary Figure 2**). The total Ashcroft score (calculated by averaging all the lobes) did not reveal any significant differences between IT and OA protocols (**Figure 1B**). The division in a frequency distribution of Ashcroft score classified as mild (0–3), moderate (=4) and severe (≥5) fibrotic lesions was applied at each time point (**Figure 1C**). This method of quantification allows to weight the contribution of each category within the final score (Stellari et al., 2017).

The frequency of severe (≥5) fibrotic lesions was higher in IT treated mice compared to OA group. Moreover, in the IT protocol group moderate and severe lesions almost equally contributed to the calculation of the total Ashcroft through the duration of the study, whereas in OA-treated mice moderate lesions were prevailing (**Figure 1C**).

As expected, the control groups treated with saline exhibited a normal lung with minimal parenchymal changes and an Ashcroft score between 0 and 3.

The homogeneous fibrotic lesion distribution observed in the OA model allowed to investigate different *ex vivo* parameters in the same mice using left lung for histology and right lobes for either hydroxyproline content or other fibrotic markers determinations. In addition, micro-CT imaging was longitudinally performed at days 7, 14, 21 and 28 for all the OA treated mice to monitor parenchymal changes.

**Figure 2:**
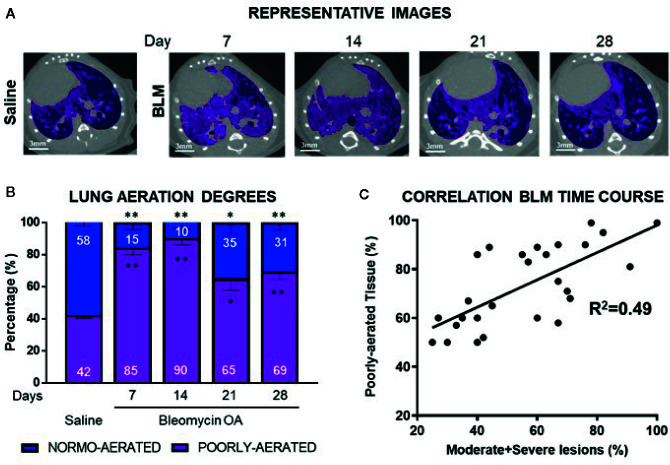
Representative lung micro-CT pictures of saline, and BLM OA mice at 7, 14, 21 and 28 days. Normally-aerated ([−900, −500] HU) and poorly-aerated ([−500, −100] HU) regions, colored in blue and pink, respectively **(A)**. Percentage of normally- and poorly-aerated tissue in BLM OA mice in the longitudinal study, from 7 to 28 days, compared to saline **(B)**. Linear correlation between 3D poorly-aerated regions and moderate and severe lesions at each time point in BLM group **(C)**. The data represent the mean ± s.e.m. of seven animals for each time point per group. Changes were compared to saline group using two-way ANOVA followed by Dunnett’s test. *p <0.05; **p <0.01.

For quantitative 3D assessment of the parenchymal lesions, clinical HU ranges were applied to semi-automatically segmented lung parenchyma distinguishing in normally-aerated ([−900, −500] HU) and poorly-aerated ([−500, −100] HU) regions (Gattinoni et al., 2001). These compartments, colored in blue and pink respectively, were defined and normalized on total lung volume (**Figure 2A**).

Representative micro-CT images at different time points clearly demonstrate that BLM OA treated mice have a homogeneous increase of the poorly-aerated tissue (**Figure 2A**), compared to saline.

Longitudinal quantification of lung aeration degrees confirmed the progressive increase in percentage of poorly-aerated tissue for BLM OA mice with a peak at 14 days (**Figure 2B**) that tended to decrease at later time points as detected by histology (**Figure 1B**).

A linear correlation for BLM group, comparing 3D quantification of poorly aerated regions by micro-CT and 2D histological frequencies of moderate and severe lesions, was performed at each time point, revealing an R^2^ = 0.49 (**Figure 2C**).

**Figure 3:**
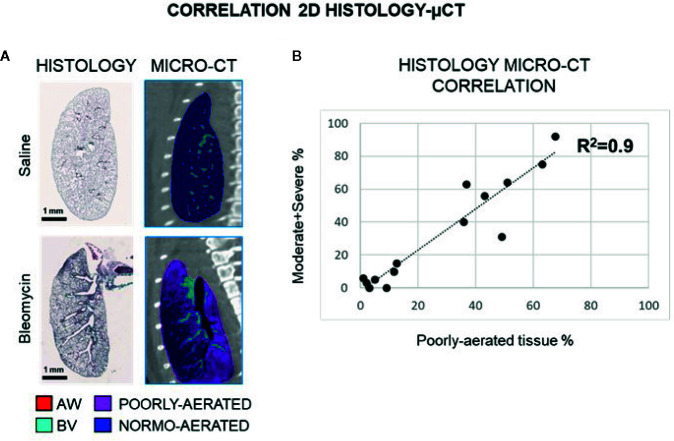
Representative histological sagittal sections and corresponding 2D micro-CT projections at 21 day **(A)**. Linear correlation between 2D poorly-aerated regions and moderate and severe lesions at each time point in saline and BLM group **(B)**.

Representative sagittal CT projections from saline and BLM OA treated mice and the corresponding histological slides are shown in **Figure 3A**, highlighting a good structural match. On these CT projections a bi-dimensional analysis was performed and the % of poorly-aerated tissue was quantified and correlated with the % of moderate and severe lesions (R^2^ = 0.9) (**Figure 3B**). The corresponding average values for BLM and saline groups are reported in **Supplementary Table 1**.

After the first set up phase, we sought to investigate the antifibrotic effect of Nintedanib (FDA approved drug) in the OA BLM mouse model by micro-CT.

Twenty-one mice in total, fourteen receiving BLM and seven treated with saline, underwent the OA protocol described above. All the mice were orally treated daily for two weeks, starting at day 7 either with vehicle or Nintedanib (60 mg/kg) (Wollin et al., 2014).

Nintedanib treatment was well tolerated and the loss of body weight was similar to BLM group (**Supplementary Figure 4A**).

At day 21, all the mice underwent micro-CT imaging and then were sacrificed to perform *ex vivo* analysis. An increase of WBC in BALF, with a predominant macrophages and lymphocytes population has been detected in the BLM group and was modestly inhibited by Nintedanib treatment (**Supplementary Figures 4B–D**).

As previously described, lungs were collected assigning the left lobe to histological analysis and right lobes to hydroxyproline determination.

**Figure 4:**
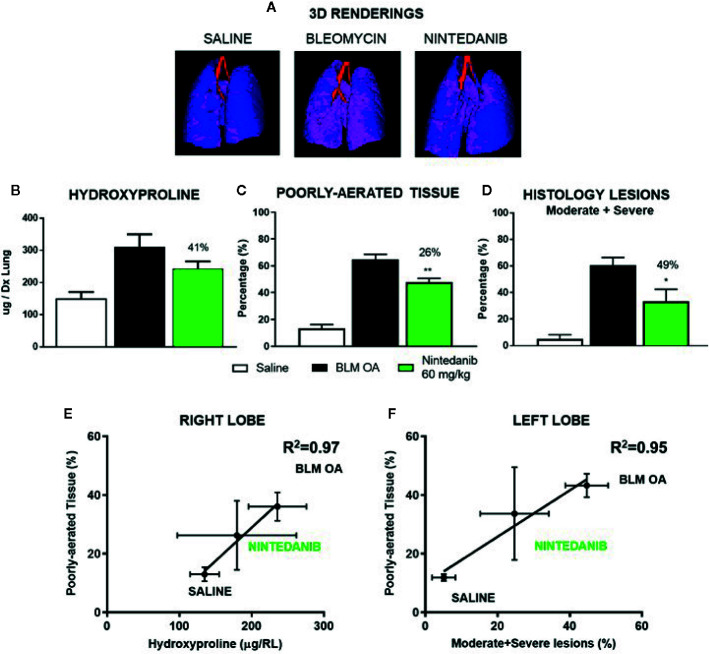
Representative 3D lungs renderings of saline, BLM and Nintedanib mice at 21 days **(A)**. Normally-aerated ([−900, −500] HU) and poorly-aerated ([−500, −100] HU) regions, colored in blue and pink, respectively. Anti-fibrotic effect of Nintedanib on: hydroxyproline concentration (right lung) **(B)**, lung aeration degrees **(C)** and percentage of moderate and severe lesions (left lung) **(D)**. Poorly-aerated area was correlated from the left and the right lobe with hydroxyproline concentration **(E)** and percentage of moderate and severe lesions **(F)**. The data represent the mean ± s.e.m. of seven animals per group. Changes were compared to the BLM group using one-way ANOVA followed by Dunnett’s test. *p <0.05; **p <0.01.

Micro-CT representative 3D lung renderings of saline, BLM and Nintedanib mice, clearly highlight the differences in tissue aeration degrees between groups (**Figure 4A**), where normally- and poorly-aerated regions have been extracted as previously described. Indeed, Nintedanib significantly inhibited the increase of poorly aerated tissue compared to BLM group (26%; p <0.01). This finding was further strengthened by *ex vivo* analysis showing that Nintedanib reduced significantly also moderate and severe fibrosis lesions (49%; p<0,05) and hydroxyproline concentration (41%) (**Figures 4B–D**). To improve the micro-CT data consistency, right and left lobes were segmented and analyzed separately. Poorly-aerated tissue was correlated with % of moderate and severe lesions (**Figure 4E**) and with hydroxyproline concentration (**Figure 4F**) for either left or right lobes, revealing R^2^ = 0.95 and R^2^ = 0.97, respectively.

## Discussion

In this study we compared two different ways of BLM administration in mice. *Ex vivo* reads out and micro-CT imaging were used to access lung fibrosis development. The overall goal was to establish a robust, reproducible pulmonary fibrosis model to use as the first screening line in drug discovery. Although in the last decade the knowledge regarding the pathogenesis of IPF has substantially improved, potential targets have been identified and two drugs received formal approval from regulatory authorities entering in the daily clinical practice, the medical need for treating this devastating disease remains very high. Many potentially effective putative anti-fibrotic compounds have been generated, but few of them overcome phase II. Pre-clinical trials are crucial steps in a drug discovery process and the selection of the best drug candidate for clinical development relies on how well experimental animal models are able to predict clinical outcomes. Different *in vivo* models have been developed over time to better understand the pathogenesis of lung fibrotic disorder, but it seems that is not sufficient (Carrington et al., 2018).

Although BLM-induced lung fibrosis models are accepted as the first line for drug testing, their clinical transability is debated. The American Thoracic Society formalized recommendations for preclinical assessment to drug development, suggesting an integrated approach using of relevant animal models, appropriate read outs for measuring evolution of lung fibrosis over time. Our experiments have highlighted several differences between IT and OA BLM administration. The heterogeneity of fibrotic lesions in IT BLM protocol better reproduces the features of the human disease. However, drug screening may result less powerful, due to the high variability in the distribution pattern. Since the precise quantification of fibrotic burden in a limited number of histological slides could be biased, larger sample sizes are needed to get the statistical significance of a given treatment. Moreover, the heterogenicity observed in the IT protocol precludes the possibility to use the same lung for different read outs, as for example histology and biochemical analysis performed on the left and on the right lobe, respectively. To overcome this issue, a common approach in preclinical studies is doubling the number of mice for experiment, albeit a direct link between fibrosis degree and other fibrosis markers is precluded.

The difference in lung fibrosis distribution between IT vs OA administration depends on the different techniques for BLM delivery. In the IT instillation technique, the solution is forced by adding a constant pressure (Stellari et al., 2012; Stellari et al., 2015a; Bergamini et al., 2017) and, for this reason, it could reach the right or the left lung randomly. Moreover, a slightly different cannula position in trachea during the instillation can influence the deposition of BLM into the airways, resulting in patchy and more severe lung lesions. We can hypothesize that for the IT BLM protocol the higher severity detected depends on the more localized distribution of the cytotoxic cascade response. This corresponds to a greater number of severe fields, compared to OA protocol. OA administration, instead, requires lower technical skills compared to IT; it is very easy to perform and benefits from the physiological breath (Barbayianni et al., 2018). By blocking the nares and preventing obligate nasal breathing, mice are forced to inhale naturally by OA aspiration and as a consequence BLM may better distribute in the whole lung, resulting in a more uniform distribution of moderate and severe lesions across lung lobes.

Our data support the evidence of homogeneous distribution of fibrotic lesions through the lung in the OA BLM model, allowing multiple read outs from the same lung as shown in the Nintedanib experiment. The OA model is fully in compliance with the main “3R principle”, Replacement, Refinement, Reduction of animal research (Flecknell, 2002; Tannenbaum and Bennett, 2015), since the number of animals can be reduced.

Although micro-CT is not able to discriminate between fibrosis and inflammation and little is known about the application of this technology for lung diseases (Vande Velde et al., 2016; Bell et al., 2018), we have shown that this tool can be used to longitudinally quantify the fibrosis in BLM OA mice using the poorly aerated tissue as a marker of disease progression. This was proved correlating the Ashcroft-based histological grading either with the 3D quantification of poorly-aerated tissue or with the same parameter extracted by the 2D analysis: the correlation coefficient markedly improved, highlighted the accuracy of micro-CT technology.

Moreover, micro-CT imaging was able to assess and quantify the antifibrotic effect of Nintedanib in BLM OA treated mice, revealing a reduction in poorly-aerated tissue comparable to the effect detected by hydroxyproline quantification and histological grading. These data strongly support the capability of micro-CT imaging in drug screening evaluation and its fully integrability with the other technologies.

## Conclusion

We strongly believe that pre-clinical models always need improvements (Wollin et al., 2014). We have proposed a new protocol for lung fibrosis development induced by double OA administration of BLM, which gives a more uniform distribution of fibrotic lesions through the lung compared to IT instillation. This new protocol presents several improvements in comparison to the existing BLM fibrosis models. It indeed turned out to be easy to perform and able to induce a fibrosis that is stable up to 4 weeks; it also exhibits two weeks of therapeutic intervention, where putative drug candidate can be administered following a therapeutic regimen (Jenkins et al., 2017; Bonniaud et al., 2018). Furthermore, one of the main advantages is to have three or more different read-outs from the same subject reducing the variability and the number of mice used, allowing a direct link between disease and biochemical analysis or molecular events.

Despite the application of micro-CT is quite new in preclinical models of lung diseases, we have found that poorly aerated tissue could be a good marker for fibrosis quantification. Micro-CT provides faster and operator-independent results compared to histological analysis, giving the possibility to monitor the disease progression in a noninvasive way since 3D anatomical information are available. We are convinced that also in preclinical studies like in the clinical setting, high-resolution CT will play a key role in providing essential information about progression and therapy of lung diseases, although, for an accurate evaluation of new therapeutic options and an improved translation of the results to human patients, guideline recommendations for post-processing analysis should be implemented (Lynch et al., 2018). As published by Sverzellati et al. (Walsh et al., 2018), in the clinical practice the use of a deep learning algorithm could help to classify high-resolution CT scans from patients with fibrotic lung disease with greater accuracy than visual assessment performed by a radiologist.

To sum up, we have demonstrated that OA model is suitable for screening anti-fibrotic compounds and that micro-CT can be fully integrated in a drug discovery process, allowing a precise and independent quantification of fibrosis progression.

Our studies paved the way for a new drug discovery approach and cast a new light on the importance to examine clinical endpoints in pre-clinical trials, giving a better indication of new anti-fibrotic drugs to push into the clinic.

## Data Availability Statement

All datasets generated for this study are included in the article/**Supplementary Material**.

## Ethics Statement

The animal study was reviewed and approved by intramural animal-welfare committee for animal experimentation of Chiesi Farmaceutici under protocol number: 449/2016-PR and comply with the European Directive 2010/63 UE, Italian D.Lgs 26/2014.

## Author Contributions

Conception and design: FRu, FS. Laboratory testing: FRu, VB, FRa, VM, Data collection: FRu, FRa, VB, VM, Data analysis and interpretation: FRu, FRa, VB, LM, LRa, NS, MS, LRu, MC, GV, FS. Drafting of manuscript: FRu, FRa, VB, LRa, GV, FS.

## Funding

This work was supported by Chiesi Farmaceutici S.p.A., that had no role in study design, data collection and analysis, decision to publish, or preparation of the manuscript.

## Conflict of Interest

FS, GV, MC, and FRu are employees of Chiesi Farmaceutici S.p.A., that supported the research work.

The remaining authors declare that the research was conducted in the absence of any commercial or financial relationships that could be construed as a potential conflict of interest.

## Supplementary Material

Figure S1 Effect of BLM administration on mice body weight. **(A)** Cellular infiltration into the bronchoalveolar lavage fluid (BALF) of mice lung, treated IT or OA with BLM and compared with saline treated control groups. Cellular infiltration was detected at 7, 14, 21 and 28 days post treatment. The amount of white blood cells (WBC) **(B)**, macrophages **(C)**, lymphocytes **(D)**, and neutrophils **(E)**, found in BALF was expressed as number of cells per microliter. The data represent the mean ± s.e.m. of seven animals per each time point per group. Changes were compared to the saline group using two-way ANOVA followed by Dunnett’s test. *p< 0.05; **p < 0.01.

Figure S2 Time course of Ashcroft score distribution in the five lobes after BLM treatment with IT or OA protocols. Left lobe (Sx); right: cranial (Dx Cr), middle (Dx M), caudal (Dx Ca), accessory lobe (Dx Ac). Data distribution of seven animals for each time point per group are graphed as box plot and the median values are linked through a light blue line. Kruskall-Wallis test was performed followed by Dunn’s multiple comparison test to compare Ashcroft score among the lung lobes. *p< 0.05; **p < 0.01.

Figure S3 Histological overview. Representative microphotographs of mice lung sections at different time points (7, 14, 21 and 28 days) after BLM treatment with IT or OA protocols and saline respectively with Masson’s trichrome stain (scale bar 250 μm).

Figure S4 Effect of Nintedanib administration on BLM OA mice body weight. **(A)** Cellular infiltration into (BALF) of BLM, BLM+Nintedanib and saline mice at day 21. The amount of white blood cells (WBC) **(B)**, macrophages **(C)**, lymphocytes **(D)** found in BALF was expressed as number of cells per microliter. The data represent the mean ± s.e.m. of seven animals per group. Changes were compared to the saline group using one-way ANOVA followed by Dunnett’s test. *p< 0.05; **p < 0.01.

Table S1 Average of micro-CT and histological parameters for BLM OA and saline groups at 21 day (data shown as mean ± s.e.m.).

## Abbreviations

BLM, bleomycin; IPF, Idiopathic pulmonary fibrosis; HRCT, highresolution computed tomography; MRI, magnetic resonance imaging; PET, positron emission tomography; IT, Intratracheal; OA, Oropharyngeal Aspiration; CT, Computed Tomography; BALF, Bronchoalveolar lavage fluid; WBC, White blood cells; HU, Hounsfield Unit; AW, Airways; BV, Blood Vessels.
