# Supplementary figures and images for: Quantification of Lung Fibrosis in IPF-Like Mouse Model and Pharmacological Response to Treatment by Micro-Computed Tomography
Source: Front Pharmacol. 2020 Jul 21;11:1117. doi: 10.3389/fphar.2020.01117 (PMC7385278; doi:10.3389/fphar.2020.01117)

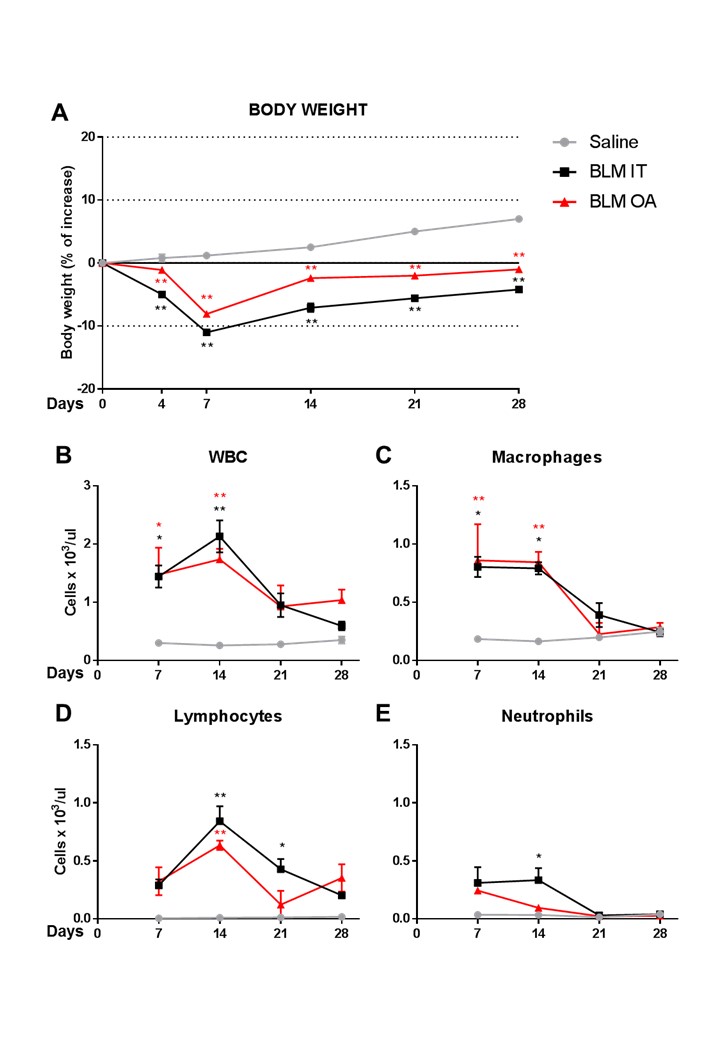

Supplement: Figure S1 — Effect of BLM administration on mice body weight. (A) Cellular infiltration into the bronchoalveolar lavage fluid (BALF) of mice lung, treated IT or OA with BLM and compared with saline treated control groups. Cellular infiltration was detected at 7, 14, 21 and 28 days post treatment. The amount of white blood cells (WBC) (B), macrophages (C), lymphocytes (D), and neutrophils (E), found in BALF was expressed as number of cells per microliter. The data represent the mean ± s.e.m. of seven animals per each time point per group. Changes were compared to the saline group using two-way ANOVA followed by Dunnett’s test. *p< 0.05; **p < 0.01. [file Image_1.jpeg]

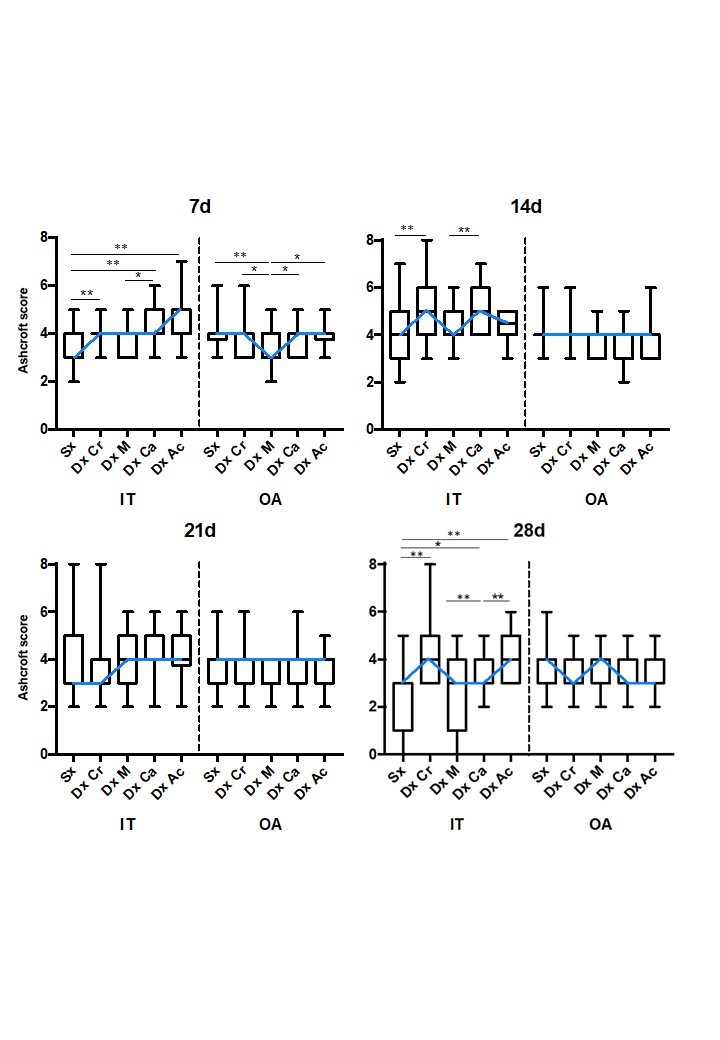

Supplement: Figure S2 — Time course of Ashcroft score distribution in the five lobes after BLM treatment with IT or OA protocols. Left lobe (Sx); right: cranial (Dx Cr), middle (Dx M), caudal (Dx Ca), accessory lobe (Dx Ac). Data distribution of seven animals for each time point per group are graphed as box plot and the median values are linked through a light blue line. Kruskall-Wallis test was performed followed by Dunn’s multiple comparison test to compare Ashcroft score among the lung lobes. *p< 0.05; **p < 0.01. [file Image_2.jpeg]

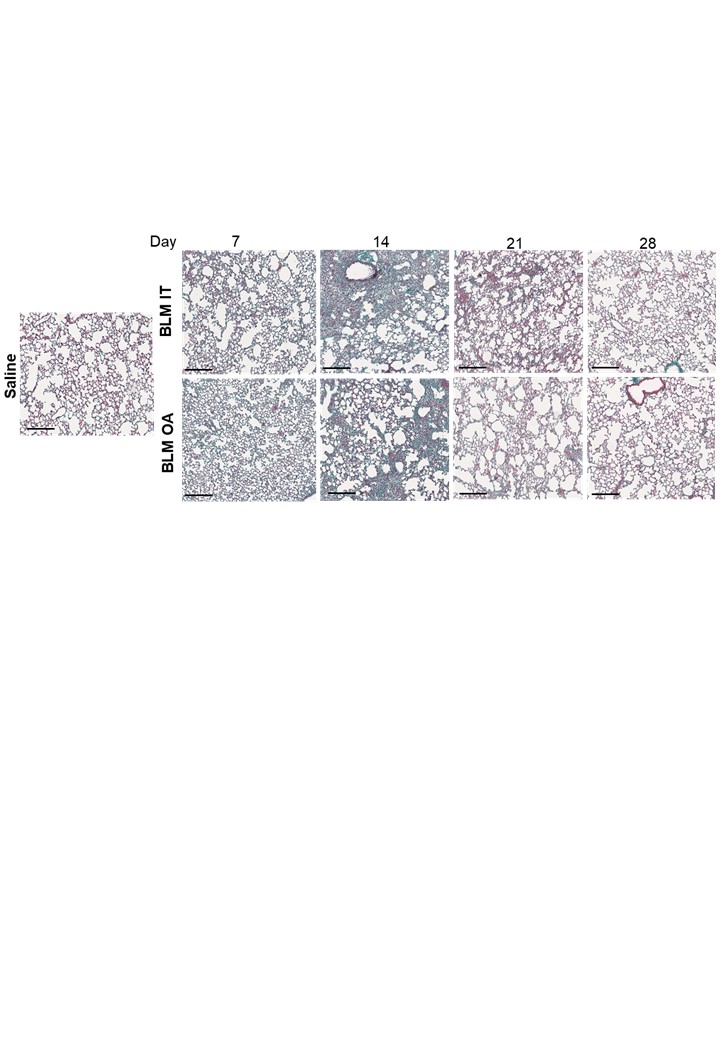

Supplement: Figure S3 — Histological overview. Representative microphotographs of mice lung sections at different time points (7, 14, 21 and 28 days) after BLM treatment with IT or OA protocols and saline respectively with Masson’s trichrome stain (scale bar 250 μm). [file Image_3.jpeg]

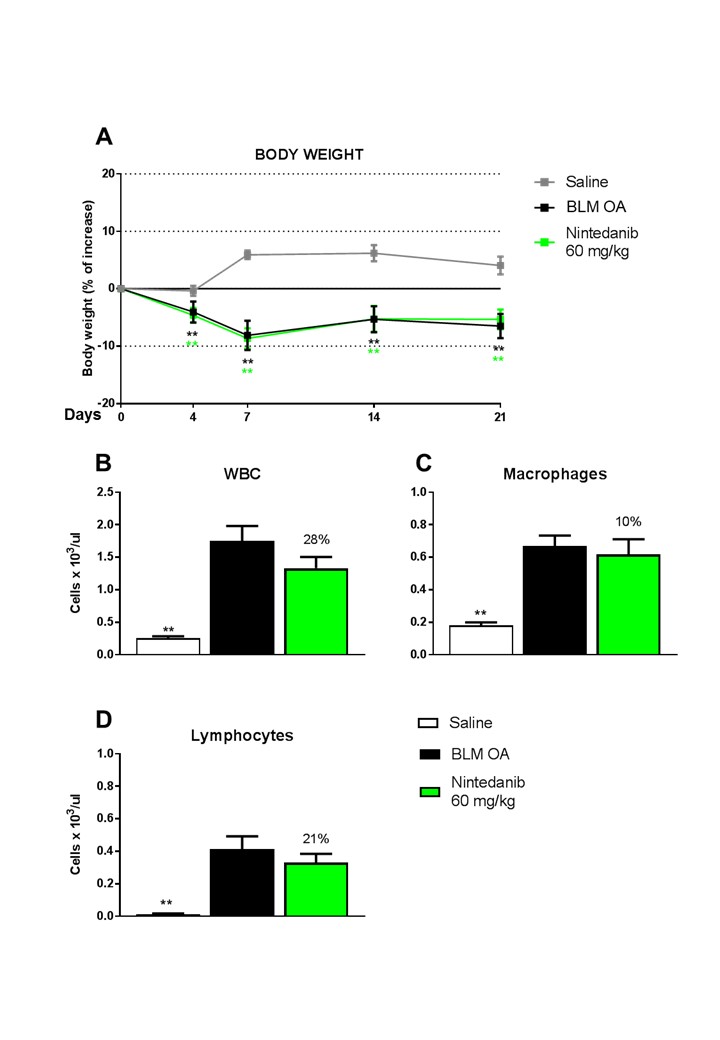

Supplement: Figure S4 — Effect of Nintedanib administration on BLM OA mice body weight. (A) Cellular infiltration into (BALF) of BLM, BLM+Nintedanib and saline mice at day 21. The amount of white blood cells (WBC) (B), macrophages (C), lymphocytes (D) found in BALF was expressed as number of cells per microliter. The data represent the mean ± s.e.m. of seven animals per group. Changes were compared to the saline group using one-way ANOVA followed by Dunnett’s test. *p< 0.05; **p < 0.01. [file Image_4.jpeg]

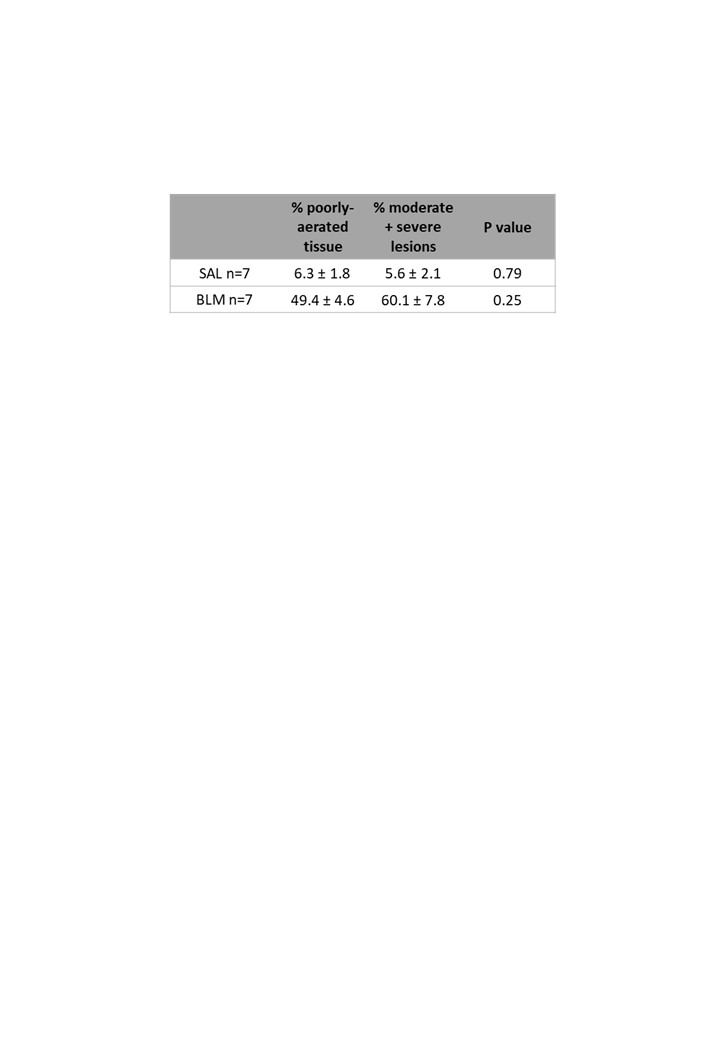

Supplement: Table S1 — Average of micro-CT and histological parameters for BLM OA and saline groups at 21 day (data shown as mean ± s.e.m.). [file Image_5.jpeg]
